# Effect of Oxygen Mole Fraction on Static Properties of Pressure-Sensitive Paint

**DOI:** 10.3390/s21041062

**Published:** 2021-02-04

**Authors:** Tomohiro Okudera, Takayuki Nagata, Miku Kasai, Yuji Saito, Taku Nonomura, Keisuke Asai

**Affiliations:** Department of Aerospace Engineering, Tohoku University, Sendai 980-8579, Japan; nagata@tohoku.ac.jp (T.N.); kasai.miku@aero.mech.tohoku.ac.jp (M.K.); yuji.saito@tohoku.ac.jp (Y.S.); nonomura@tohoku.ac.jp (T.N.); keisuke.asai.c3@tohoku.ac.jp (K.A.)

**Keywords:** pressure-sensitive paint, oxygen mole fraction, optimal condition

## Abstract

The effects of the oxygen mole fraction on the static properties of pressure-sensitive paint (PSP) were investigated. Sample coupon tests using a calibration chamber were conducted for poly(hexafluoroisopropyl methacrylate)-based PSP (PHFIPM-PSP), polymer/ceramic PSP (PC-PSP), and anodized aluminum PSP (AA-PSP). The oxygen mole fraction was set to 0.1–100%, and the ambient pressure ($P_{\mathrm{ref}}$) was set to 0.5–140 kPa. Localized Stern–Volmer coefficient $B_{\mathrm{local}}$ increased and then decreased with increasing oxygen mole fraction. Although $B_{\mathrm{local}}$ depends on both ambient pressure and the oxygen mole fraction, its effect can be characterized as a function of the partial pressure of oxygen. For AA-PSP and PHFIPM-PSP, which are low-pressure- and relatively low-pressure-type PSPs, respectively, $B_{\mathrm{local}}$ peaks at $P_{O_{2}\mathrm{ref}}<12$ kPa. In contrast, for PC-PSP, which is an atmospheric-pressure-type PSP in the investigated range, $B_{\mathrm{local}}$ does not have a peak. $B_{\mathrm{local}}$ has a peak at a relatively high partial pressure of oxygen due to the oxygen permeability of the polymer used in the binder. The peak of $S_{\mathrm{PR}}$, which is the emission intensity change with respect to normalized pressure fluctuation, appears at a lower partial pressure of oxygen than that of $B_{\mathrm{local}}$. This is because the intensity of PSP becomes quite low at a high partial pressure of oxygen even if $B_{\mathrm{local}}$ is high. Hence, the optimal oxygen mole fraction depends on the type of PSP and the ambient pressure range of the experiment. This optimal value can be found on the basis of the partial pressure of oxygen.

## 1. Introduction

The measurement of the distributions of state quantities such as pressure, density, and velocity in a fluid dynamic field is important for the performance evaluation of fluid machines and the clarification of flow dynamics. Therefore, aerodynamic measurement techniques are essential tools. One of the most important measurement targets is surface pressure distribution. However, complex models that have many pressure taps and tubes are required for the acquisition of pressure distributions. For small or thin test models, it is extremely difficult to carry out multi-point measurements. Pressure-sensitive paint (PSP) [1] is an effective tool for measuring surface pressure distributions with high spatial resolution without pressure tubes in wind tunnel models. Pressure measurements using PSP are achieved by measuring the intensity or lifetime of PSP emissions under a constant oxygen mole fraction. PSP is composed of oxygen-sensitive dye molecules, which are quenched via interactions with oxygen molecules, and a binder that fixes the dye molecules to model surfaces. The characteristics of PSP depend on the combination of dye molecules, binder, additives, and other components.

Aerodynamic measurements and flow visualization using PSP have been conducted for various measurement targets in various conditions [2,3,4,5,6,7,8]. Instantaneous measurements in particular have been actively conducted and developed in recent years [9,10].

The range of ambient pressure is a critical parameter in PSP measurements because it has a large impact on the characteristics of PSP, such as pressure sensitivity and emission intensity. Anyoji et al. [11,12] conducted PSP measurements on a flat plate in compressible low-Reynolds-number conditions. They acquired a time-averaged pressure distribution at an ambient pressure of 2 to 20 kPa. Time-resolved PSP measurements under low-pressure and subsonic conditions are more difficult. Nagata et al. [13] conducted PSP measurements on the surface of a circular cylinder at several kPa. They extracted the characteristic modes of pressure fluctuation from a large number of time-series PSP images with a low signal-to-noise ratio (SNR) by applying randomized singular value decomposition [14]. Niimi et al. [15] applied PSP for measurements in the high-Knudsen-number regime, which includes rarefied gas flow mainly at pressures lower than 150 Pa. Mitsuo [16] investigated the characteristics of PSP in the ultra-low-pressure range of $10^{-3}$–$10^{2}$ Pa.

Aerodynamic measurements in flows at high (up to real-flight) Reynolds numbers are conducted at high pressure (>100 kPa) to increase the Reynolds number [17]. A critical issue for PSP measurements at high pressure is the relatively low intensity of PSP emission. For cryogenic wind tunnels, low-pressure-type PSP, such as anodized aluminum PSP (AA-PSP) and poly(1-trimethylsilyl-1-propyne) PSP (PTMSP-PSP), are used even though the wind tunnel working gas is pressurized [18,19,20] because the oxygen mole fraction of the working gas is quite low (approximately 0.1%). The characteristics of PSP are affected by the oxygen mole fraction even if the ambient pressure is fixed. These characteristics have been investigated under lower oxygen mole fractions. Asai et al. and Sakaue et al. [18,21] developed AA-PSP for cryogenic wind tunnels. They investigated the characteristics of various kinds of AA-PSP via sample coupon tests and clarified the influence of the oxygen mole fraction on pressure sensitivity at 100 K in the oxygen mole fraction range of 4 to 2000 ppm and stagnation pressure range of 0.4 to 190 kPa. Asai et al. [19] developed PTMSP-PSP for cryogenic and unsteady tests. They conducted sample coupon tests at cryogenic temperatures and low oxygen mole fractions of less than 0.1% and applied the developed PSP in wind tunnel experiments. Egami et al. [20] determined the appropriate combination of dye molecules and binder for PSP measurements under cryogenic temperature and low oxygen mole fractions of less than 0.1%. Ono et al. [22] conducted sample coupon tests in a mixture of CO${}_{2}$ and O${}_{2}$ with an oxygen mole fraction of 0.1% for PSP measurements in a wind tunnel that simulated the atmospheric environment of Mars.

Oglesby et al. [23] derived equations for the relative error in measured pressure by PSP as a function of normalized intensity of PSP emission, the relative error in pressure as a function of pressure, and the relationship between sensitivity and pressure based on the Stern–Volmer equation. Their analysis provided the fundamental limits on the achievable sensitivity and accuracy in the considered model. Nagata et al. [24] evaluated the optimal pressure range of several types of PSP in air using several criteria. They showed that the optimal pressure range can be obtained from the Stern–Volmer coefficient or the emission intensity change with respect to normalized pressure fluctuation.

Because PSP is a sensor that utilizes oxygen quenching, the optimal pressure should correspond to the optimal partial pressure of oxygen (or the oxygen mole fraction). Although the characteristics of PSP under oxygen mole fractions different from that of air have been investigated, the effect of the oxygen mole fraction on the characteristics of PSP in a wide range of conditions has not been examined. In the present study, the effect of the oxygen mole fraction on the static properties of PSP is investigated. Sample coupon tests are conducted for poly(hexafluoroisopropyl methacrylate)-based PSP (PHFIPM-PSP), polymer/ceramic PSP (PC-PSP), and AA-PSP under several oxygen mole fractions in a wide range of ambient pressures. The static properties are evaluated on the basis of Stern–Volmer coefficient *B*, which is commonly used in the wind tunnel community, localized Stern–Volmer coefficient $B_{\mathrm{local}}$, and $S_{\mathrm{PR}}$, which is the emission intensity change with respect to normalized pressure fluctuation. The aim of the present study is to improve PSP measurements in severe conditions by tuning the oxygen mole fraction of the working gas.

## 2. Principal of PSP and Evaluation Parameters

### 2.1. Principal of PSP

The pressure sensitivity of PSP is based on oxygen quenching. It theoretically obeys the Stern–Volmer equation:

(1) $$\frac{I_{0}}{I(P,T)}=1+K\left(T\right)P,$$

where

(2) $$K\left(T\right)=\varphi_{O_{2}}S\left(T\right)K_{\mathrm{SV}}.$$

Here, $I_{0}$ and *I* are the intensities of PSP emissions in the oxygen-free condition and at pressure *P*, respectively; S(T) is the solubility coefficients, which depend on binder temperature *T*; and K(T) and $K_{\mathrm{SV}}$ are the Stern–Volmer coefficients for the ambient pressure and oxygen mole fraction $\varphi_{O_{2}}$, respectively. In common wind tunnel experiments, Equation (1) is normalized by the reference condition ($P_{\mathrm{ref}}$ and $I_{\mathrm{ref}}$), which is generally the wind-off condition, because it is difficult to produce conditions without oxygen. Normalizing Equation (1) by the ambient pressure (absolute pressure) and the intensity in the wind-off condition ($I_{\mathrm{ref}}$ at $P_{\mathrm{ref}}$) yields the following equation:

(3) $$\frac{I_{\mathrm{ref}}}{I(P,T)}=A\left(T\right)+B\left(T\right)\left(\frac{P}{P_{\mathrm{ref}}}\right),$$

where

(4) $$\left\{\begin{array}{c} A\left(T\right)=\frac{1}{1+K\left(T\right)P_{\mathrm{ref}}},\\ B\left(T\right)=\frac{K\left(T\right)P_{\mathrm{ref}}}{1+K\left(T\right)P_{\mathrm{ref}}}. \end{array}\right.$$

Here, A(T) and B(T) are Stern–Volmer coefficients commonly used in the wind tunnel community. These coefficients are temperature-dependent because of thermal quenching.

The ideal behavior of PSP is described by the linear model in Equation (3); however, practical PSP exhibits nonlinearity. Therefore, Equation (3) is rewritten as $I_{\mathrm{ref}}/I=f\left(P/P_{\mathrm{ref}}\right)$ for practical PSP, where *f* is a nonlinear function. The following equation, which is similar to Equation (3), is obtained from the first-order Taylor series approximation:

(5) $$\frac{I_{\mathrm{ref}}}{I}=A_{\mathrm{local}}+B_{\mathrm{local}}\frac{P}{P_{\mathrm{ref}}},$$

where $A_{\mathrm{local}}$ and $B_{\mathrm{local}}$ are localized Stern–Volmer coefficients. The characteristics of the tested PSP are mainly discussed on the basis of this equation. $B_{\mathrm{local}}$ and its related parameters are used as evaluation parameters (see the following subsection). In addition, Equations (3) and (5) can be rewritten as a function of pressure coefficient $C_{P}$, Mach number *M*, and specific heat ratio γ in the variable-pressure condition in wind tunnel experiments as follows [24]:

(6) $$\frac{I_{\mathrm{ref}}}{I}=A_{\mathrm{local}}+B_{\mathrm{local}}\left(1+\frac{\gamma }{2}M^{2}C_{P}\right).$$

Here, $C_{P}$ is the pressure coefficient defined as

(7) $$C_{P}=\frac{P-P_{\infty }}{1/2\rho_{\infty }u_{\infty }^{2}}=\frac{P-P_{\mathrm{ref}}}{1/2\rho_{\infty }u_{\infty }^{2}},$$

where $\rho_{\infty }$ and $u_{\infty }$ are the density and velocity in the mainstream in wind tunnel experiments, respectively. This equation indicates that $I_{\mathrm{ref}}/I$ does not explicitly depend on the ambient pressure $P_{\mathrm{ref}}$, but on the Mach number and the $C_{p}$ distribution. It should be noted that although the order of $C_{p}$ is almost constant, its distribution is affected by ambient pressure $P_{\mathrm{ref}}$ via the Reynolds number.

### 2.2. Evaluation Parameters

The characteristics of PSP are evaluated using two parameters introduced by Nagata et al. [24]. These parameters evaluate the performance of PSP in terms of pressure fluctuations due to fluid phenomena. The first parameter is $B_{\mathrm{local}}$, which corresponds to normalized pressure sensitivity.

(8) $$B_{\mathrm{local}}\equiv {\left.\frac{d\{I\left(P_{\mathrm{ref}}\right)/I\left(P\right)\}}{d(P/P_{\mathrm{ref}})}\right|}_{P=P_{\mathrm{ref}}}.$$

Under a constant oxygen mole fraction, we can evaluate the PSP sensitivity using $B_{\mathrm{local}}$ (or *B* for ideal PSP) regardless of the influence of the ambient pressure on the magnitude of the pressure fluctuation caused by fluid phenomena because $I_{\mathrm{ref}}/I$ does not depend on $P_{\mathrm{ref}}$ (Equation (6)). The magnitude of the intensity changes due to pressure fluctuation is also important in instantaneous measurements such as single-shot lifetime-based measurements and time-resolved measurements. The second parameter is $S_{\mathrm{PR}}$. This parameter indicates the emission intensity change with respect to pressure fluctuation ΔP, which is proportional to ambient pressure. $S_{\mathrm{PR}}$ is defined on the basis of Equation (5) as

(9) $$S_{\mathrm{PR}}\equiv -{\left.\frac{dI}{dP}\right|}_{\Delta P=0}={\left.\frac{I_{\mathrm{ref}}B_{\mathrm{local}}}{{\left(A_{\mathrm{local}}+B_{\mathrm{local}}P\right)}^{2}}\right|}_{\Delta P=0}=I_{\mathrm{ref}}B_{\mathrm{local}},$$

where $P=\left(P_{\mathrm{ref}}+\Delta P\right)/P_{\mathrm{ref}}=P/P_{\mathrm{ref}}$, and thus the pressure fluctuation is normalized by the ambient pressure, as done for $B_{\mathrm{local}}$. This parameter indicates the gradient of the $P/P_{\mathrm{ref}}$–*I* curve.

For a given ambient pressure, larger values of these parameters are more advantageous for measurements. The optimal ambient pressure in terms of maximizing these evaluation parameters can be derived analytically for the linear model in Equation (3). Parameter $B_{\mathrm{local}}$ corresponds to *B* in the linear model, and $S_{\mathrm{PR}}$ becomes the following:

(10) $$\begin{array}{cc} S_{\mathrm{PR}}\equiv & {\left.-\frac{dI}{dP}\right|}_{\Delta P=0}=I_{\mathrm{ref}}B=\frac{I_{0}KP_{\mathrm{ref}}}{{\left(1+KP_{\mathrm{ref}}\right)}^{2}}. \end{array}$$

From Equation (4), the optimal ambient pressure in terms of maximizing *B* is $P_{\mathrm{ref}}=\infty$. The optimal ambient pressure in terms of maximizing $S_{\mathrm{PR}}$ can be obtained by finding the pressure at which the derivative of $S_{\mathrm{PR}}$ becomes zero using Equation (10):

(11) $$0=\frac{dS_{\mathrm{PR}}}{dP_{\mathrm{ref}}}=\frac{d}{dP_{\mathrm{ref}}}\left\{\frac{I_{0}KP_{\mathrm{ref}}}{{\left(1+KP_{\mathrm{ref}}\right)}^{2}}\right\}=\frac{I_{0}K\left(1-KP_{\mathrm{ref}}\right)}{{\left(1+KP_{\mathrm{ref}}\right)}^{3}},$$

and thus $S_{\mathrm{PR}}$ is maximized at $P_{\mathrm{ref}}=1/K$. Oglesby et al. [23] discussed the optimal pressure in terms of minimizing the relative error in measured pressure $\epsilon =\frac{\Delta P_{\epsilon }}{P}$. They derived such a condition by finding the pressure at which the derivative of ϵ becomes zero. Here, $\Delta P_{\epsilon }$ is the error in measured pressure *P* due to the uncertainty in measured PSP emission $\Delta \left(I/I_{0}\right)$. Based on the leading term of the Taylor expansion of Equation (1) with respect to *P*, the small finite error in pressure due to uncertainty in the measured PSP emission is

(12) $$\begin{array}{c} \Delta P_{\epsilon }\approx \frac{{\left(1+KP\right)}^{2}}{K}\Delta \left(I/I_{0}\right), \end{array}$$

and the relative error in the measured pressure can be written as

(13) $$\begin{array}{cc} \epsilon =\frac{\Delta P_{\epsilon }}{P_{\mathrm{ref}}}\approx & \frac{{\left(1+KP_{\mathrm{ref}}\right)}^{2}}{KP_{\mathrm{ref}}}\Delta \left(I/I_{0}\right), \end{array}$$

where $P\approx P_{\mathrm{ref}}$. Therefore, the optimal ambient pressure in terms of minimizing the relative error in the measured pressure can be obtained as

(14) $$\begin{array}{cc} 0= & \frac{d\epsilon }{dP_{\mathrm{ref}}}\approx \frac{d}{dP_{\mathrm{ref}}}\left\{\frac{{\left(1+KP_{\mathrm{ref}}\right)}^{2}}{KP_{\mathrm{ref}}}\Delta \left(I/I_{0}\right)\right\}=\frac{\left(K^{2}-\frac{1}{P_{\mathrm{ref}}^{2}}\right)}{K}\Delta \left(I/I_{0}\right), \end{array}$$

and the optimal ambient pressure is $P_{\mathrm{ref}}=1/K$. The relative error in the measured pressure fluctuation caused by fluid phenomena is also minimized at $P_{\mathrm{ref}}=1/K$ because the magnitude of the pressure fluctuation is proportional to the ambient pressure. Ambient pressure $P_{\mathrm{ref}}$ values at which the parameters in the linear model become maximized and minimized are summarized in Table 1.

These optimal conditions are based on the linear model under a constant oxygen mole fraction. Therefore, the optimal pressure may change due to changes in the oxygen mole fraction and the nonlinearity in the characteristics of PSP.

## 3. Experimental Setup

**Figure 1:**
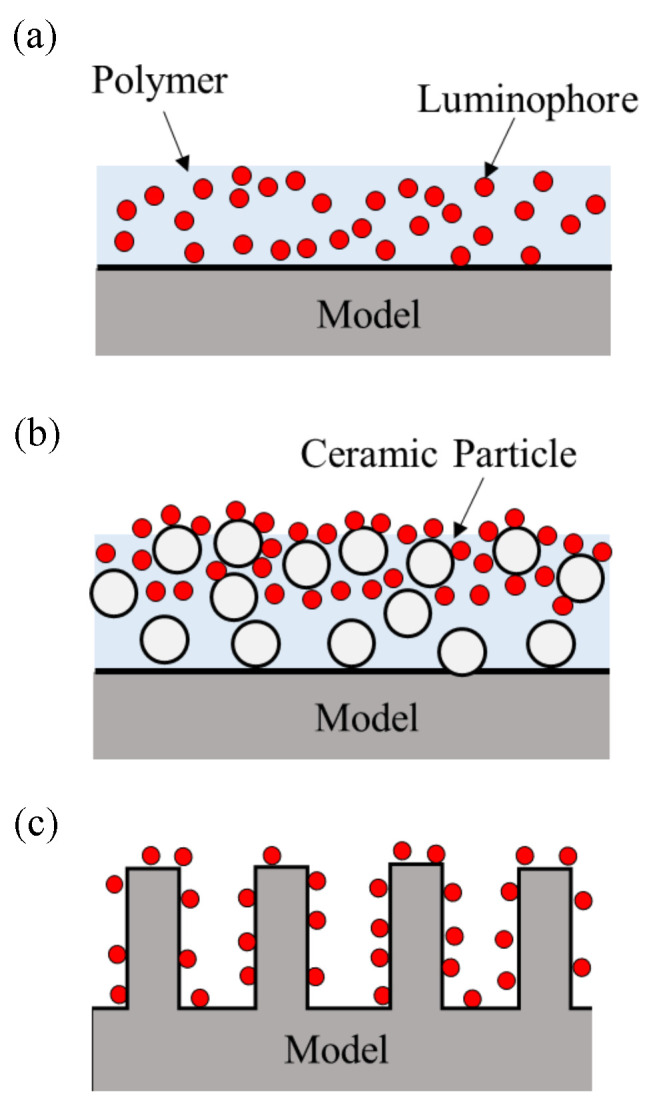
Schematic diagrams of the binder structure of (**a**) PHFIPM-PSP, (**b**) PC-PSP, and (**c**) AA-PSP.

Sample coupon tests using three kinds of PSP with different binder structures that are widely used in wind tunnel tests, namely PHFIPM-PSP [25], PC-PSP [26], and AA-PSP [21,27], were conducted in a calibration chamber. The compositions of the tested PSPs are given in Table 2. Schematic diagrams of the PSP structures are shown in Figure 1. The binder of PHFIPM-PSP is composed of polymer. Dye molecules dispersed in PHFIPM. This paint is a relatively low-pressure-type PSP and is suitable for steady-state measurements. PC-PSP and AA-PSP are atmospheric-pressure- and low-pressure-type PSPs. The binder of PC-PSP is a mixture of ceramic particles and a polymer. The dye molecules are in the near-surface region. The binder of AA-PSP is anodized aluminum. The dye molecules are on its surface. Here, the dye molecules of AA-PSP are Ru(dpp)${}_{3}$; PtTFPP is rarely used as a dye in AA-PSP.

The sample coupon tests were conducted under oxygen mole fractions ($\varphi_{O_{2}}$) of 0.1%, 1%, 10%, 21%, 40%, and 100%. The pressure inside the calibration chamber was varied in the ranges of $P_{\mathrm{ref}}=0.5$ to 30 kPa and $P_{\mathrm{ref}}=60$ to 140 kPa, while the temperature of the sample coupon was maintained at 293 K. The lower pressure range follows a previous study [24] that showed that PSP exhibits interesting behavior below $P_{\mathrm{ref}}=30$ kPa in air. The higher pressure range was included because the partial pressure of oxygen decreases at a lower oxygen mole fraction. Sample coupons were prepared for each oxygen mole fraction to mitigate the influence of photodegradation. For PHFIPM-PSP and PC-PSP, the paint was applied onto the surface of each sample coupon at the same time. The procedure for fabricating AA-PSP was as follows. A single large degreased sample was anodized in dilute sulfuric acid at 283 K for 30 min. The current density was 12.5 mA/cm${}^{2}$. After anodization, the sample was soaked in phosphoric acid at 298 K for 20 min. The sample was then dried in a desiccator and cut into six pieces. Finally, each piece was dipped in the dye solution for 10 s.

**Figure 2:**
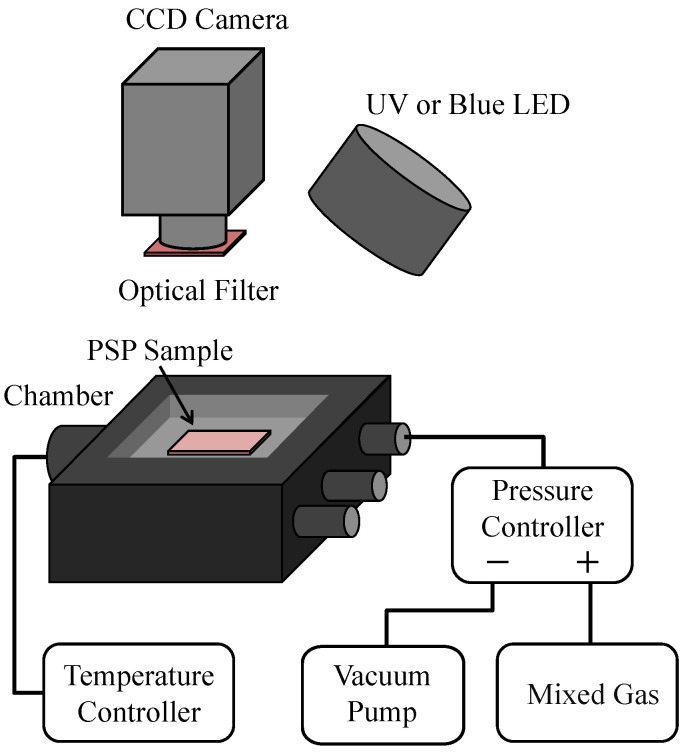
Schematic diagram of the calibration chamber.

A schematic diagram of the calibration chamber is shown in Figure 2. The pressure in the calibration chamber was controlled using a pressure controller (DPI 515, Druck, Leicester, England). The temperature of the sample coupons was controlled using a temperature controller with a Peltier element (MT886-D1000, NetsuDenshi Kogyo, Tokyo, Japan ). The precision of the pressure and temperature controllers was 30 Pa and 0.05 K, respectively. For oxygen mole fractions other than 21%, a mixed gas cylinder with the given oxygen mole fraction (Taiyo Nippon Sanso Corporation, Tokyo, Japan) was used as the high-pressure source for the pressure controller. The oxygen mole fraction in these cylinders was guaranteed by the supplier. Compressed dry air was used for the oxygen mole fraction of 21%. The compositions of the gases in these cylinders are listed in Table 3. A 16-bit charge-coupled device (CCD) camera (ORCA II-BT1024, Hamamatsu Photonics, Shizuoka, Japan) with a camera lens (Nikkor 105 mm f/2.8, Nikon, Tokyo, Japan) was used as a photodetector. For PHFIPM-PSP and PC-PSP, an ultraviolet light-emitting diode (UV-LED; IL-106, Hardsoft, Kraków, Poland) with a center wavelength of 395 nm was used as the excitation light source, and an optical filter (650±20 nm, PB0650/040, Asahi Spectra, Tokyo, Japan) was mounted in front of the camera lens. For AA-PSP, a blue LED (LEDA294-470) with a center wavelength of 470 nm was used as the excitation light source, and an optical filter (640±50 nm, PB0640/100, Asahi Spectra, Tokyo, Japan) was mounted in front of the camera lens. The exposure time of the camera was set to achieve a PSP emission intensity of approximately 90% of the full-well capacity at the minimum ambient pressure for each oxygen mole fraction. One image was obtained in each case, and the emission intensity was averaged over at least 200×200 pixels except for the edges of the sample coupon. It should be noted that the pressure in the chamber and the temperature of the sample coupon were accurately controlled by the pressure and temperature controllers, respectively, and thus the variation in the time direction was negligible.

Although the airtightness of the chamber is very high, the gas in the chamber was exchanged twice for every pressure condition, and the effect of leakage was reduced as much as possible. The pressure of the gas was changed using a multi-step process. For example, it was changed from 10 kPa to 12 kPa as follows: 10 kPa→1 kPa→150 kPa→1 kPa→150 kPa→12 kPa. In this way, the chamber was always filled with the gas supplied from the cylinder, and the oxygen mole fraction was precisely controlled. A mechanical shutter was installed in the calibration chamber to mitigate the influence of photodegradation. The sample coupon was excited only at the moment of imaging. Photodegradation correction was conducted via linear interpolation in the time direction using images acquired at the start and end of the calibration tests for each oxygen mole fraction.

The uncertainty of $B_{\mathrm{local}}$ was calculated from the error propagation. This uncertainty stems from errors related to the camera and pressure controller. The errors related to the camera include dark current noise, readout noise, and shot noise. Because the background is subtracted, the former two sources can be ignored. The maximum uncertainty of $B_{\mathrm{local}}$ in the present study was approximately 5% at $P_{\mathrm{ref}}=1$ kPa; the uncertainty decreased with increasing ambient pressure. For example, the uncertainties at $P_{\mathrm{ref}}=10$ and 100 kPa were approximately 2% and 0.6%, respectively.

## 4. Results and Discussion

**Figure 3:**
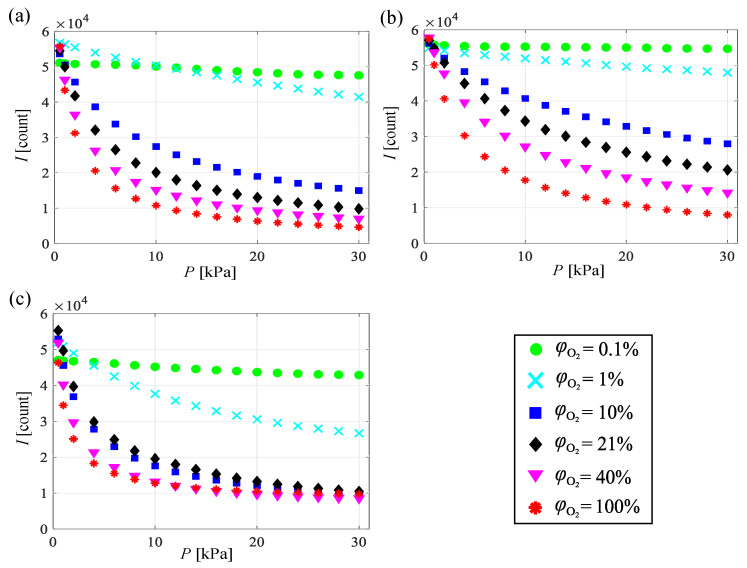
Intensity of PSP emission at various oxygen mole fractions for (**a**) PHFIPM-PSP, (**b**) PC-PSP, and (**c**) AA-PSP.

Figure 3 shows the change in the emission intensity of PHFIPM-PSP, PC-PSP, and AA-PSP versus the ambient pressure in the calibration chamber at T=293 K for various oxygen mole fractions. The PSP emission intensity decreased as the ambient pressure increased due to enhanced oxygen quenching. The oxygen mole fraction greatly affected the gradient of the *P*–*I* curves. This gradient becomes large (small) at high (low) oxygen mole fractions because the change in the partial pressure of oxygen corresponding to the number of oxygen molecules in the binder at the steady state depends on the oxygen mole fraction. The difference in the change rate of the emission intensity of the PSPs is related to pressure sensitivity. PSP measurements at high oxygen mole fractions, especially measurements with a limit on the exposure time such as time-resolved measurements, are difficult due to the resulting small emission intensity.

**Figure 4:**
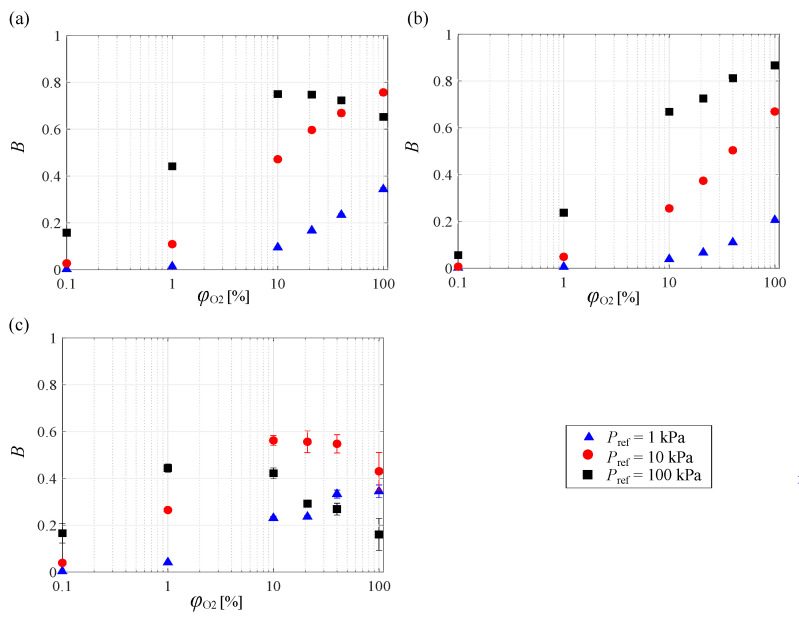
Effect of oxygen mole fraction on *B* in various pressure ranges for (**a**) PHFIPM-PSP, (**b**) PC-PSP, and (**c**) AA-PSP.

Figure 4 shows the effects of the oxygen mole fraction on *B* at various pressure ranges. Here, *B* was computed via the linear (PHFIPM-PSP and PC-PSP) or quadratic (AA-PSP) fitting of the Stern–Volmer curves ($P/P_{\mathrm{ref}}$–$I_{\mathrm{ref}}/I$ curves) for each oxygen mole fraction. The pressure ranges used for calculating *B* at $P_{\mathrm{ref}}=1$ kPa, 10 kPa, and 100 kPa were 0.5 to 10 kPa, 0.5 to 20 kPa, and 60 to 140 kPa, respectively. As a rough trend, *B* increased with increasing oxygen mole fraction, and the baseline of *B* increased with increasing ambient pressure. However, the detailed trend was different for each PSP and ambient pressure range. For all PSPs, *B* at $P_{\mathrm{ref}}=1$ kPa continuously increased with increasing oxygen mole fraction. This trend was the same at $P_{\mathrm{ref}}=10$ and 100 kPa for PC-PSP and at $P_{\mathrm{ref}}=10$ kPa for PHFIPM-PSP. However, in other cases, such as PHFIPM-PSP at $P_{\mathrm{ref}}=100$ kPa and AA-PSP at $P_{\mathrm{ref}}=10$ and 100 kPa, *B* had a peak for each ambient pressure. This result indicates that the PSP measurement of fluid phenomena improved at appropriate oxygen mole fractions at a certain ambient pressure in the tested pressure range.

Changes in the oxygen mole fraction correspond to the change in ambient pressure at a constant oxygen mole fraction in terms of the partial pressure of oxygen. Therefore, there is an optimal oxygen mole fraction (partial pressure of oxygen), just as there is an optimal ambient pressure in air.

Low and high oxygen mole fractions correspond to low and high ambient pressures for air. Hence, for AA-PSP, which has a high *B* at low ambient pressure in air, *B* decreased with increasing oxygen mole fraction at low ambient pressure ($P_{\mathrm{ref}}=10$ kPa) at $\varphi_{O_{2}}>21\%$. In contrast, for PC-PSP, which has a high *B* at around atmospheric pressure in air, *B* did not decrease in the investigated ranges of the oxygen mole fraction and ambient pressure. However, for PC-PSP, *B* decreased with increasing oxygen mole fraction at very high ambient pressure.

**Figure 5:**
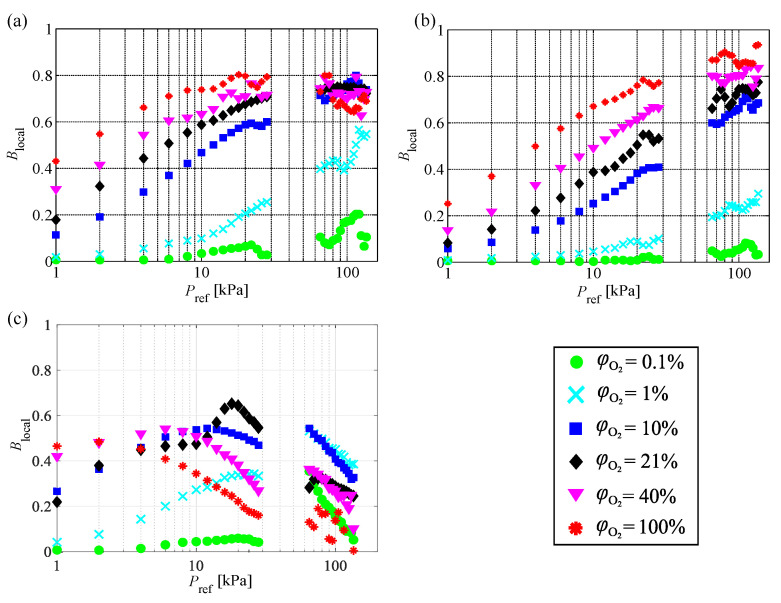
Effect of ambient pressure on $B_{\mathrm{local}}$ at various oxygen mole fractions for (**a**) PHFIPM-PSP, (**b**) PC-PSP, and (**c**) AA-PSP.

Figure 5 shows the influence of the ambient pressure on $B_{\mathrm{local}}$ for several oxygen mole fractions. For PC-PSP, as a rough trend, $B_{\mathrm{local}}$ increased with increasing ambient pressure at each oxygen mole fraction. $B_{\mathrm{local}}$ also increased with increasing oxygen mole fraction at each ambient pressure, as discussed for Figure 4. This is because the partial pressure of oxygen increased with increasing ambient pressure or oxygen mole fraction at each oxygen mole fraction. Trends similar to those discussed for Figure 4 were observed for PHFIPM-PSP and AA-PSP.

**Figure 6:**
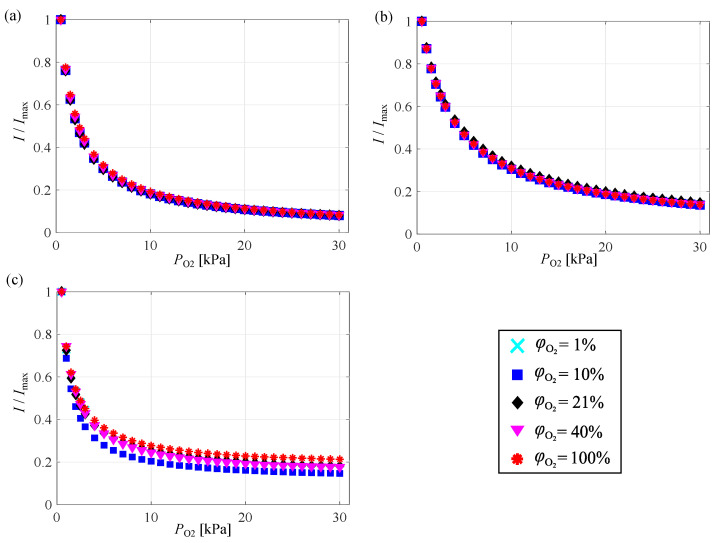
Normalized intensity of PSP emission versus partial pressure of oxygen at various oxygen mole fractions for (**a**) PHFIPM-PSP, (**b**) PC-PSP, and (**c**) AA-PSP.

Figure 6 shows the intensities of PSP emission normalized by the maximum intensity at each oxygen mole fraction. The normalized intensity of PSP emission decreased with increasing partial pressure of oxygen. Unlike in Figure 3, the normalized intensity at different oxygen mole fractions can be characterized by the partial pressure of oxygen. For more information on the variation of AA-PSP, please refer to the Appendix A.

**Figure 7:**
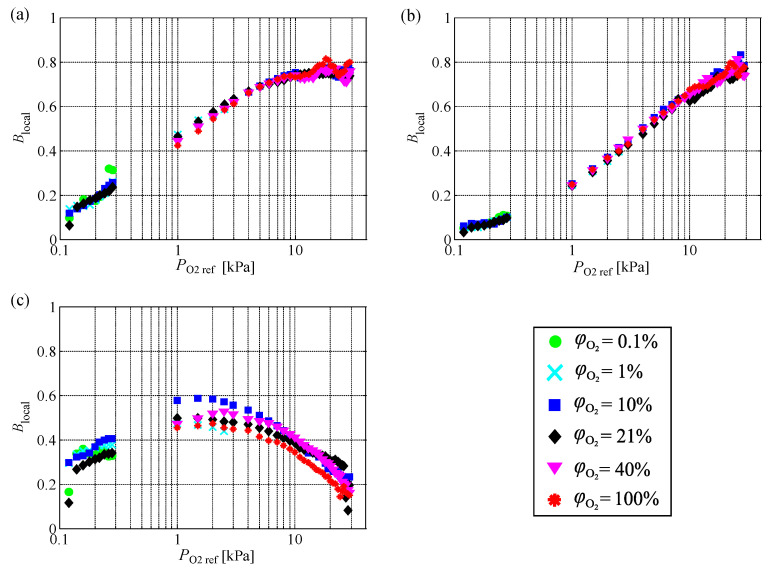
Effect of partial pressure of oxygen on $B_{\mathrm{local}}$ at various oxygen mole fractions for (**a**) PHFIPM-PSP, (**b**) PC-PSP, and (**c**) AA-PSP.

Figure 7 shows the effect of the oxygen mole fraction on $B_{\mathrm{local}}$ as a function of the partial pressure of oxygen $P_{O2\mathrm{ref}}$. The gaps in the figure are caused by the limits of the pressure controller. The maximum partial pressure of oxygen in the test of $\varphi_{O_{2}}=0.1$% was 0.3 kPa, and the minimum partial pressure of oxygen in the test of $\varphi_{O_{2}}=100$% was 0.5 kPa. Hence, the dataset has gaps. Curves of $B_{\mathrm{local}}$ for different oxygen mole fractions can be characterized by the partial pressure of oxygen. The values of $B_{\mathrm{local}}$ for all PSPs increased with increasing oxygen mole fraction at a low partial pressure of oxygen. In the tested condition, $B_{\mathrm{local}}$ had a peak, except for PC-PSP. For PHFIPM-PSP, the partial pressure of oxygen at which $B_{\mathrm{local}}$ peaked was approximately 20 kPa. This peak appeared at a much lower partial pressure of oxygen for AA-PSP. Although there was no peak at the tested partial pressures of oxygen for PC-PSP, it may appear at a much higher partial pressure of oxygen.

The partial pressure of oxygen at which $B_{\mathrm{local}}$ (and *B*) peaked reflects the characteristics of PSP. For PC-PSP, a relatively high partial pressure of oxygen is required to dissolve oxygen molecules into the binder because the binder consists of an ester polymer, in which the solubility of oxygen is low, and TiO${}_{2}$ particles. The solubility of oxygen in PHFIPM, the binder of PHFIPM-PSP, is higher than that in the polymer used in PC-PSP, and thus more oxygen dissolves at a given partial pressure of oxygen. When oxygen is sufficiently dissolved in the binder and becomes saturated, the aspect of oxygen quenching is less likely to change with respect to changes in the surrounding partial pressure of oxygen, and as a result, $B_{\mathrm{local}}$ decreases with increasing partial pressure of oxygen. This behavior occurs at a relatively low pressure or oxygen mole fraction when the solubility of oxygen is high. For AA-PSP, the dye molecules are on the surface of the anodized aluminum binder. Because the dye molecules are exposed directly to the ambient gas, $B_{\mathrm{local}}$ decreases even though the partial pressure of oxygen is quite low. Hence, the existence of an optimal partial pressure of oxygen depends on the binder structure of PSP.

Here, the effect of the oxygen mole fraction observed in the experiments is compared to that derived from the linear model. According to the Smoluchowski relation, the Stern–Volmer coefficient for the pressure (*K*) of a polymer-based PSP can be modeled as follows [1]:

(15) $$K=4\pi R_{\mathrm{AB}}N_{0}D\varphi_{O_{2}}S,$$

where $R_{\mathrm{AB}}$ is the interaction distance between the dye molecules and oxygen molecules, $N_{0}$ is the Avogadro constant, and *D* is the diffusivity. On the basis of Equations (1)–(4) and (15), *B* for the ideal polymer-based PSP can be characterized by the partial pressure of oxygen $P_{O_{2}\mathrm{ref}}$, solubility of oxygen *S*, and diffusivity *D*. *B* increases with increasing partial pressure of oxygen, solubility of oxygen, and diffusivity because *K* increases. This is related to the pressure at which *B* peaks being $P_{\mathrm{ref}}=\infty$, as shown in Table 1. This trend qualitatively agrees with the present experimental results up to the partial pressure of oxygen at which $B_{\mathrm{local}}$ peaks. However, $B_{\mathrm{local}}$ decreases at a higher partial pressure of oxygen because the oxygen in the binder saturates. The partial pressure of oxygen at which $B_{\mathrm{local}}$ peaks is considered to depend on the solubility of oxygen and diffusivity (i.e., oxygen permeability). For example, for a binder with high oxygen permeability, more oxygen molecules are taken into the binder and saturation occurs even at a small oxygen partial pressure. In this case, the $B_{\mathrm{local}}$ peak appears at a lower partial pressure of oxygen.

The present results show that the change in $B_{\mathrm{local}}$, including the effect of the saturation of oxygen in the binder, can be described by the partial pressure of oxygen. The same explanation holds true for AA-PSP at the typical temperature described by the collision-controlled model. The partial pressure of oxygen at which $B_{\mathrm{local}}$ peaks is considered to be affected by the binder structure such as pore diameter. The partial pressure of oxygen at which $B_{\mathrm{local}}$ peaks is lower than that of polymer-based PSP because the dye molecules are on the surface of the binder and exposed to the atmosphere.

**Figure 8:**
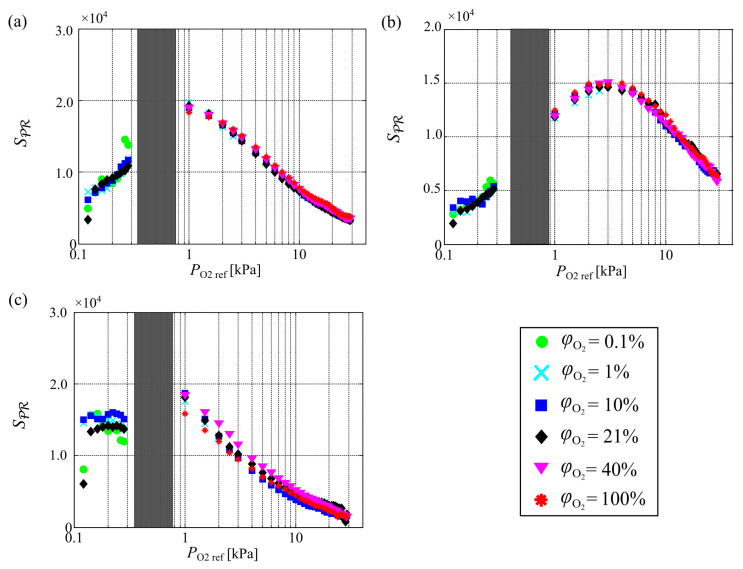
Effect of partial pressure of oxygen on $S_{\mathrm{PR}}$ at various oxygen mole fractions for (**a**) PHFIPM-PSP, (**b**) PC-PSP, and (**c**) AA-PSP.

Figure 8 shows the effect of the partial pressure of oxygen on $S_{\mathrm{PR}}$ at various oxygen mole fractions. Here, $S_{\mathrm{PR}}$ includes information regarding the intensity of PSP emission. The optical setup was changed for each PSP and between $P_{O_{2}\mathrm{ref}}<0.3$ kPa and $P_{O_{2}\mathrm{ref}}\geq 1.0$ kPa, and thus the values of $S_{\mathrm{PR}}$ cannot be compared between PSPs and between $P_{O_{2}\mathrm{ref}}<0.3$ kPa and $P_{O_{2}\mathrm{ref}}\geq 1.0$ kPa. With increasing oxygen mole fraction, $B_{\mathrm{local}}$ increases up to a certain partial pressure of oxygen, as discussed previously. However, the intensity of the PSP emission decreases with increasing partial pressure of oxygen. It is one of the critical issues because the smaller intensity of PSP emission decreases the SNR. Therefore, the intensity change with pressure changes is an important property. As with $B_{\mathrm{local}}$, the effect of the oxygen mole fraction on $S_{\mathrm{PR}}$ can be characterized by the partial pressure of oxygen. The partial pressure of oxygen at which $S_{\mathrm{PR}}$ peaks depends on the PSP. A high $B_{\mathrm{local}}$ and a high intensity are required to increase $S_{\mathrm{PR}}$. Hence, $S_{\mathrm{PR}}$ peaks at a lower partial pressure of oxygen than that of $B_{\mathrm{local}}$.

## 5. Conclusions

In the present study, we investigated the effect of the oxygen mole fraction on the static properties of three kinds of PSP, namely PHFIPM-PSP, PC-PSP, and AA-PSP, using sample coupon tests. The oxygen mole fraction was set to 0.1–100%, and the ambient pressure was set to 0.5–140 kPa. The static characteristics of the PSPs were evaluated using Stern–Volmer coefficient *B*, localized Stern–Volmer coefficient $B_{\mathrm{local}}$, and $S_{\mathrm{PR}}$, which is the emission intensity change with respect to normalized pressure fluctuation.

The obtained data indicate that the intensity of PSP emission normalized by the maximum intensity at each oxygen mole fraction can be characterized by the partial pressure of oxygen. For PHFIPM-PSP and AA-PSP, *B* and $B_{\mathrm{local}}$ initially increase with increasing oxygen mole fraction and then decrease. Hence, there is an optimal mole fraction, the value of which depends on the ambient pressure for each type of PSP. The Stern–Volmer curve can be characterized by the partial pressure of oxygen, and thus the optimal condition can be found based on the partial pressure of oxygen regardless of ambient pressure. The partial pressure of oxygen at which $B_{\mathrm{local}}$ peaks is considered to be related to the structure of the binder (e.g., oxygen permeability for polymer-based PSP). Because the saturation of the oxygen in the binder occurs even though the partial pressure of oxygen is low, the partial pressure of oxygen at which $B_{\mathrm{local}}$ peaks is low when the oxygen permeability is high. For AA-PSP, the partial pressure of oxygen at which $B_{\mathrm{local}}$ peaks is lower than that for the polymer-based PSPs. This is considered to be related to the characteristics of the binder (i.e., dye molecules are on the surface of the binder). The effect of the oxygen mole fraction on $S_{\mathrm{PR}}$ was also investigated. This parameter involves both $B_{\mathrm{local}}$ and the intensity of PSP emission, and thus its peak appears at a lower partial pressure of oxygen than that of $B_{\mathrm{local}}$.

Localized Stern–Volmer coefficient $B_{\mathrm{local}}$ indicates the change in the normalized intensity of PSP emission versus normalized pressure. Therefore, the optimal oxygen mole fraction can be determined on the basis of the relationship between $B_{\mathrm{local}}$ and the partial pressure of oxygen when the intensity is not important such as the situation, which the on-tip accumulation of photon is valid. The magnitude of the intensity change due to pressure fluctuation is important when an instantaneous measurement is required (e.g., single-shot lifetime-based measurements and time-resolved measurements). For such measurements, the optimal oxygen mole fraction can be determined on the basis of the relationship between the partial pressure of oxygen and $S_{\mathrm{PR}}$.

The present results show that a higher oxygen mole fraction is better for measurements using atmospheric-pressure-type PSPs, such as PC-PSP, at low pressure, whereas a lower oxygen mole fraction is better for measurements using low-pressure-type PSPs, such as PHFIPM-PSP and AA-PSP, at atmospheric and higher pressure.

The effect of oxygen mole fraction on temperature characteristics of PSP was not investigated in the present study. This topic may need to be studied in the future.

## Figures and Tables

**Table 1:** Optimal ambient pressure for each parameter in the linear model.

Parameter	Maximum Cond.	Minimum Cond.
Stern–Volmer coefficient *B*	*∞*	0
Intensity change with normalized pressure fluctuation $S_{\mathrm{PR}}$	1/K	0,∞
Pressure sensitivity $S_{P}$ [1/kPa] [23]	0	*∞*
Relative error in measured pressure ϵ [23]	0,∞	1/K

**Table 2:** Composition of tested PSPs.

	PHFIPM-PSP [25]	PC-PSP [26]	AA-PSP [21,27]
Binder	PHFIPM	Ester polymer TiO${}_{2}$ (rutile)	Anodized aluminum
Dye	PtTFPP	PtTFPP	Ru(dpp)${}_{3}$
Solvent	Ethyl acetate	Toluene (binder) Toluene, methanol (dye)	Dichloromethane
Remarks	Low-temperature sensitivity Relatively low-pressure type	Fast response Atmospheric-pressure type	Fast response Low-pressure type

**Table 3:** Composition of cylinder gas for various oxygen mole fractions.

Oxygen mole fraction	0.1%	1%	10%	21%	40%	100%
O${}_{2}$	0.0990%	0.996%	10.00%	21%	40.00%	>99.7%
Other components	N${}_{2}$	N${}_{2}$	N${}_{2}$	N${}_{2}$, Ar, CO${}_{2}$, etc	N${}_{2}$	N${}_{2}$, Ar

**Figure A1:**
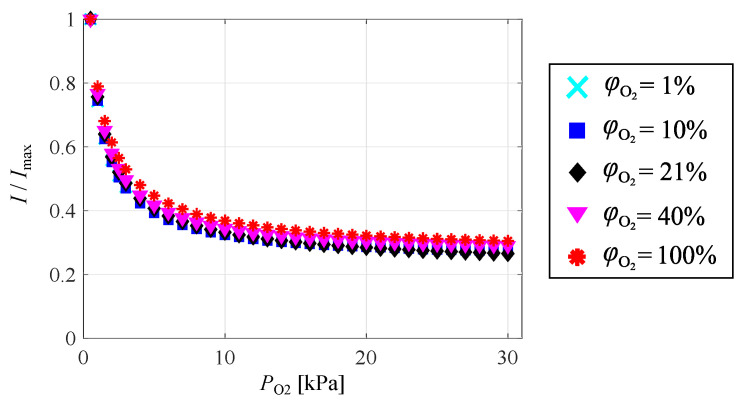
Normalized emission intensity of AA-PSP versus partial pressure of oxygen at various oxygen mole fractions ($\varphi_{O_{2}}$) tested using a single sample coupon.

## Data Availability

Not applicable.

## Appendix A

Figure 6 shows that the normalized emission intensity of AA-PSP was not be completely characterized by the partial pressure of oxygen. We investigated whether this was due to individual differences in sample coupons. A single sample coupon was tested at all oxygen mole fractions. Considering degradation with time, the test was conducted twice, with the sequence of oxygen mole fractions reversed for the second test.

Figure A1 shows the normalized emission intensity of AA-PSP averaged for the two tests. Degradation with time was compensated for. The trend for the nonaveraged results of each experiment was the same as that for the averaged results, which shows that the effects of degradation with time are weak. The results without the effects of individual differences in sample coupons still show that the variation cannot be completely characterized by the partial pressure of oxygen. This issue will be further investigated in a future study.
